# The Microbiota–Gut–Brain Axis in Autism: Associations, Causal Inference, and Interventions—A Narrative Review

**DOI:** 10.3390/pathogens14111145

**Published:** 2025-11-11

**Authors:** Zhiqiang Zhang, Wenkai Kang, Yu Mi, Xin Zhong, Yulong He

**Affiliations:** 1 Tomas Lindahl Nobel Laureate Laboratory, The Seventh Affiliated Hospital, Sun Yat-sen University, Shenzhen 518107, China; zhangzhq79@mail2.sysu.edu.cn (Z.Z.); kangwk@mail2.sysu.edu.cn (W.K.); miyauomm@163.com (Y.M.); 2 Digestive Diseases Center, Guangdong Provincial Key Laboratory of Digestive Cancer Research, The Seventh Affiliated Hospital, Sun Yat-sen University, Shenzhen 518107, China

**Keywords:** autism spectrum disorder, Brain–Gut Axis, microbiota, metabolism, Signs and Symptoms, Digestive

## Abstract

Autism spectrum disorder is markedly heterogeneous and frequently accompanied by gastrointestinal symptoms that often correlate with behavioral phenotypes. Emerging evidence suggests that the microbiota–gut–brain axis may contribute to these associations through multiple bidirectional communication routes—including neural, immune, and endocrine pathways, as well as microbial metabolites such as short-chain fatty acids and tryptophan–kynurenine intermediates. This narrative review synthesizes clinical, mechanistic, and interventional evidence published between January 2010 and July 2025, clarifies the extent to which current data support association versus causation, evaluates key confounding factors, summarizes evidence for interventions such as probiotics, prebiotics, and fecal microbiota transplantation, and outlines future directions for precision research and targeted interventions based on functional pathways and stratified subgroups.

## 1. Introduction

### 1.1. Autism Spectrum Disorder (ASD) Heterogeneity and Epidemiologic Challenges

ASD represents a highly heterogeneous neurodevelopmental condition characterized by persistent deficits in social communication and interaction, alongside restricted and repetitive behaviors, interests, or activities [1,2]. Its etiology is multifactorial: genetic susceptibility interacts with diverse environmental and biological influences, including immune–inflammatory pathways [3,4] and oxidative stress [5]. No single pathophysiological mechanism fully accounts for the marked phenotypic variability [6,7,8]. Epidemiologic surveillance over the past decade indicates a global rise in ASD prevalence, underscoring its public health significance [9]. Current interventions primarily alleviate symptoms and enhance functioning, while disease-modifying therapies remain unavailable [10,11]. Accordingly, identifying modifiable biological and environmental pathways that contribute to ASD-related phenotypes represents a critical research priority [12,13].

### 1.2. The Microbiota–Gut–Brain Axis (MGBA) Framework for Understanding ASD

The MGBA describes a bidirectional communication network between the gut and the central nervous system (CNS), mediated by intestinal microbiota [14,15]. This network operates through multiple interacting routes [16]: (1) neural—primarily via the vagus nerve (VN), enteric nervous system (ENS), and sympathetic nervous system (SNS); (2) immune—through innate and adaptive immune cells and cytokine signaling; (3) endocrine—via the hypothalamic–pituitary–adrenal (HPA) axis; and (4) metabolic—through microbially derived metabolites such as short-chain fatty acids (SCFAs) and tryptophan derivatives that can modulate glial activity and neural circuits. This framework offers a conceptual model for exploring how gut physiology may influence brain function and behavior. Nevertheless, current findings are largely correlational, providing mechanistic hypotheses rather than demonstrating causality [17].

### 1.3. Gastrointestinal Comorbidity in ASD: Prevalence and Clinical Correlates

Multiple studies indicate that gastrointestinal (GI) symptoms (e.g., constipation, diarrhea, abdominal pain, and bloating) are more prevalent among individuals with ASD [18,19,20]. Reported prevalence varies across studies due to differences in assessment tools, age groups, recruitment settings, and medication use, but meta-analyses estimate that roughly one-third of individuals with ASD are affected—significantly higher than in the general pediatric population [21]. The presence and severity of GI symptoms frequently correlate with greater social communication deficits, restricted and repetitive behaviors, anxiety, and sleep disturbances [22]. However, these associations remain correlational, and causal relationships are not yet established. This pattern highlights the MGBA as a potentially informative framework for investigating the biological underpinnings of ASD’s clinical heterogeneity [23].

## 2. Methods and Search Strategy

This article is a narrative review, structured with a systematic search strategy to ensure methodological transparency. The literature selection process was twofold:

First, a primary systematic search was conducted using the PubMed database for articles published in the modern era of MGBA research, defined as 1 January 2010, through 31 July 2025. The search utilized a keyword combination of (“Autism spectrum disorder”) AND (“Brain-Gut Axis” OR “Microbiota” OR “Metabolism” OR “Signs and Symptoms, Digestive”). This primary search identified 859 articles.

Second, to provide essential historical context, this systematic search was supplemented by an expert-driven identification of foundational, pre-2010 studies that were identified through the authors’ existing knowledge and citation tracking from key review articles.

The final set of 146 publications was curated from both the systematically identified pool (the 859 articles) and the supplemental foundational pool. The selection was guided by the following criteria. Inclusion criteria were as follows: (1) articles published in English; (2) original research (human or key mechanistic animal models) or review articles; (3) studies directly addressing the MGBA in the context of ASD; and (4) high relevance to the review’s core themes of mechanistic pathways, methodological challenges, causal inference, or therapeutic interventions. Exclusion criteria were as follows: (1) articles not relevant to the core themes; (2) editorials, letters, or case reports (unless highly illustrative); and (3) studies primarily focused on non-ASD conditions.

Figures were created with BioRender (www.biorender.com, accessed on 27 September 2025). The conceptual frameworks in Box 1 and Box 2 were designed by the authors. A generative AI assistant (ChatGPT 4.0) was used only for text refinement and formatting. The authors directed and verified all steps and take full responsibility for the scientific accuracy of the figures and boxes.

Box 1 **Dissecting Bidirectional Causality: A Framework for Forward and Reverse Inference.**

Establishing a robust causal link between the microbiota and ASD requires a framework that can not only validate the forward pathway (microbiome → ASD) but also rigorously assess the reverse pathway (ASD → microbiome), accounting for critical confounders like behavior and diet.

**Part A: Validating the Microbiome → ASD Pathway (Forward Causality)**

This three-step roadmap translates genetically prioritized signals into biologically validated mechanisms.

- **Step 1: Prioritize Causal Candidates with MR and Colocalization.**
  ○ **Action:** Employ robust MR analyses and colocalization to identify microbial functions or metabolites with a putative causal link to ASD, ensuring shared genetic causality.
  ○ **Outcome:** A high-confidence list of candidates for experimental validation.

- **Step 2: Interrogate Mechanisms in Patient-Derived Functional Models.**
  ○ **Action:** Expose prioritized metabolites to patient-derived intestinal or BBB organoids.
  ○ **Outcome:** Quantify key endpoints like barrier integrity (TEER, tight junctions) and immune activation (cytokine profiles) to establish biological plausibility.

- **Step 3: Confirm Causality with Dose-Response and Time-Series Analyses.**
  ○ **Action:** Perform systematic dose-response and time-series experiments. Where feasible, use inhibitors for reverse validation.
  ○ **Outcome:** Robust preclinical evidence of a specific, dose-dependent causal relationship.

**Part B: Investigating the ASD → Microbiome Pathway (Reverse Causality)**

This step is critical for disentangling whether microbial alterations are causes or consequences of ASD-related phenotypes.

- **Action 1: Employ Bidirectional MR.**
  ○ **Method:** Use genetic instruments for ASD as the exposure and microbial features as the outcome. This formally tests the hypothesis that genetic liability for ASD causally influences the gut microbiome.
  ○ **Outcome:** Genetic evidence for or against a causal effect of ASD on the microbiome, helping to untangle the direction of the primary association.

- **Action 2: Implement Longitudinal and Cross-Lagged Models.**
  ○ **Method:** In prospective cohort studies, use advanced statistical models (e.g., cross-lagged panel models, linear mixed-effects models) to analyze the temporal interplay between ASD-related behaviors (e.g., changes in diet, medication use) and microbiome shifts over time.
  ○ **Outcome:** A dynamic, longitudinal understanding of how ASD-related factors drive microbial changes, thereby statistically controlling for the confounding effects discussed throughout this review.

Box 2 **Major Confounders and Practical Control Strategies in MGBA–ASD Research.**

**Dietary factors.** Dietary selectivity and restricted food variety are common in ASD and strongly influence gut microbial composition. Future studies can apply structured 24 h dietary recalls or food-frequency questionnaires, with energy adjustment or macronutrient normalization, to mitigate dietary bias.

**Medication exposure.** Antibiotics, proton pump inhibitors (PPIs), and psychotropic medications can induce lasting shifts in gut microbial ecology. Recording medication history and applying stratified analyses—or incorporating medication washout periods where ethically feasible—can reduce pharmacological confounding.

**Comorbidities.** Gastrointestinal disorders, anxiety, and sleep disturbances frequently co-occur with ASD and may independently affect MGBA signaling. Sensitivity analyses that exclude or statistically adjust for these conditions help clarify independent microbiome–behavior associations.

**Sample collection and processing.** Variability in sampling time, dietary state (fasting vs. postprandial), and storage conditions introduces noise. Harmonized collection protocols—standardizing sampling timing, pre-sampling diet, and biospecimen handling—enhance reproducibility and cross-cohort comparability.

**Overall,** acknowledging and systematically addressing these confounders will strengthen causal inference and the translational reliability of MGBA-related findings in ASD research.

## 3. MGBA: Bidirectional Neurobiology

**Figure 1:**
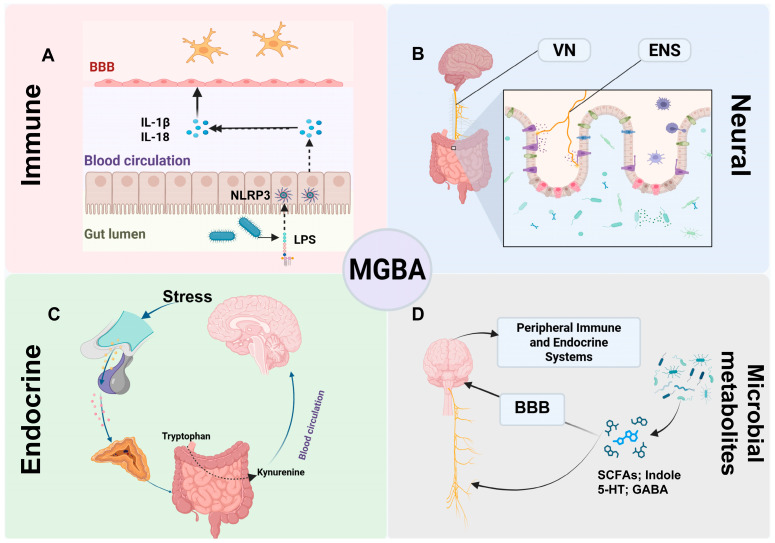
**Overview of bidirectional MGBA modules and their crosstalk. Flow Explanation:** This schematic illustrates the four principal communication routes constituting the MGBA and their reciprocal interactions. (**A**) Immune module: Gut dysbiosis or external stimuli allow LPS to activate NLRP3 inflammasomes, promoting IL-1β/IL-18 release and BBB disruption, linking peripheral inflammation to neuroinflammation. (**B**) Neural module: The VN and ENS transmit microbial, hormonal, and immune signals from the gut to the brain, providing a rapid neural conduit for bidirectional regulation. (**C**) Endocrine module: Stress activates the HPA axis, altering tryptophan metabolism toward the kynurenine pathway, thereby influencing neuroimmune signaling. (**D**) Microbial metabolite module: Microbial products act through endocrine and immune routes or interact with the BBB to modulate neural and systemic homeostasis. Together, these interconnected pathways form a dynamic network through which gut microbes influence brain function and behavior, and vice versa. **Abbreviations**: MGBA, microbiota–gut–brain axis; BBB, blood–brain barrier; IL-1β, interleukin-1 beta; IL-18, interleukin-18; NLRP3, NLR family pyrin domain-containing 3 (inflammasome); LPS, lipopolysaccharide; VN, vagus nerve; ENS, enteric nervous system; SCFAs, short-chain fatty acids; 5-HT, 5-hydroxytryptamine (serotonin); GABA, γ-aminobutyric acid.

The MGBA encompasses four interacting modules—neural, immune, endocrine, and microbial metabolic—that communicate through systemic circulation and across the blood–brain barrier (BBB) (Figure 1) [16].

### 3.1. Immune: Barrier, Cytokines, and Neuroinflammation

Integrity of the intestinal barrier is essential for maintaining host–microbiota symbiosis and homeostasis [24]. When dysbiosis or external factors such as diet compromise this barrier, pathogen-associated molecular patterns, including lipopolysaccharide (LPS), can translocate across the mucosa into circulation, activating innate immune pathways such as the NLRP3 inflammasome and inducing interleukin-1β (IL-1β) and interleukin-18 (IL-18) release [25,26,27]. Systemic inflammation may subsequently disrupt BBB integrity through cytokine signaling and loss of endothelial tight junctions, thereby promoting neuroinflammation. Microglial activation and polarization are consistently reported in ASD models and human studies [28,29,30,31]. Recent evidence also implicates central NLRP3 activation in BBB dysfunction. In ASD, in vivo imaging and histological analyses reveal abnormal microglial activation [32]. Nonetheless, most evidence remains correlational and insufficient to infer causality [26,33,34].

### 3.2. Neural: A Rapid Conduit

Because the enteric (ENS) and sympathetic (SNS) nervous systems are less extensively studied, this section focuses on the VN. Among MGBA pathways, the VN provides the most rapid anatomical and physiological link between the gut and the CNS [35]. As the tenth cranial nerve and the main afferent–efferent branch of the parasympathetic system, the VN descends from the brainstem to the thoracoabdominal cavity, extensively innervating visceral organs, including the gastrointestinal tract [36]. Approximately 80% of its fibers are afferent, establishing a predominantly bottom-up flow of information from the gut to central nodes such as the nucleus tractus solitarius [37,38,39]. Peripheral VN terminals detect luminal and mucosal signals—including microbial metabolites, gut-derived hormones, and inflammatory mediators—and relay them to the brainstem–limbic–cortical network, modulating emotional and cognitive processing [35,37,40,41]. Classic animal studies demonstrate that *Bifidobacterium longum* NCC3001 induces anxiolytic-like effects in mice with chronic colitis, effects abolished after vagotomy, underscoring the VN’s role in microbiota-to-brain signaling [42,43]. The VN also regulates peripheral immunity via the cholinergic anti-inflammatory reflex and the sympathetic–splenic pathway, reinforcing its function as an integrative hub within the MGBA [44].

### 3.3. Endocrine: HPA Axis

The endocrine system connects stress and metabolic states to the MGBA through the HPA axis [45,46]. Stress-induced cortisol elevations upregulate the rate-limiting enzymes tryptophan 2,3-dioxygenase (TDO) and indoleamine 2,3-dioxygenase (IDO), shifting tryptophan metabolism toward the kynurenine pathway and reducing serotonin synthesis [47]. Kynurenine metabolites, in turn, modulate neuroimmune signaling and affective behaviors. Evidence from human and animal studies supports these mechanisms, though effect sizes and population variability remain to be clarified [48,49].

### 3.4. Systems Crosstalk: From Pathways to Metabolites

MGBA signaling operates through parallel, interconnected neural (vagal and enteric), immune (NLRP3–cytokine), endocrine (HPA), and metabolic (SCFAs and tryptophan–kynurenine) pathways. For instance, peripheral LPS-induced inflammation can alter the excitability of vagal nodose ganglion neurons [50,51]. Microbiota-derived metabolites are discussed in Section 3.

## 4. Microbial Metabolites: Regulatory Roles in Neural Function

Within the MGBA, microbial metabolites act as key mediators linking gut ecology with host physiology. Table 1 summarizes their major classes, representative sources, mechanisms, and effects on neural function. These metabolites influence the brain through multiple routes: some cross the BBB, others act on endothelial receptors, modulate peripheral immune or endocrine signaling, or affect brain networks via the vagus nerve. They reflect the functional state of the microbiota and represent potential intervention targets, although current evidence remains largely correlational and mechanistic.

### 4.1. SCFAs: Gut-to-Brain Mediators

Short-chain fatty acids—mainly acetate, propionate, and butyrate—are produced by anaerobic fermentation of dietary fiber and resistant starch, and their levels can be modulated by altering the gut microbiota through probiotic intake [52,56,57,58,59]. Butyrate serves as a key energy source for colonic epithelial cells, upregulating tight-junction proteins (e.g., claudin, occludin) and maintaining barrier integrity [60], thereby limiting bacterial translocation into the circulation.

A subset of SCFAs crosses the BBB via monocarboxylate transporters [61,62], or acts on free fatty acid receptor 3 on brain endothelial cells [63,64,65], modulating BBB stress responses and permeability. Centrally, SCFAs regulate microglial maturation, homeostasis, and neuroinflammation [66]; butyrate additionally inhibits histone deacetylases [67,68,69] and is associated with increased brain-derived neurotrophic factor expression, synaptogenesis, and neurogenesis in preclinical models [52]. Overall, SCFAs link diet, microbiota, barrier function, and neural plasticity, representing intervention targets that require further validation in well-controlled human studies [70,71,72].

### 4.2. Neuroactive Compounds and Precursors

**Figure 2:**
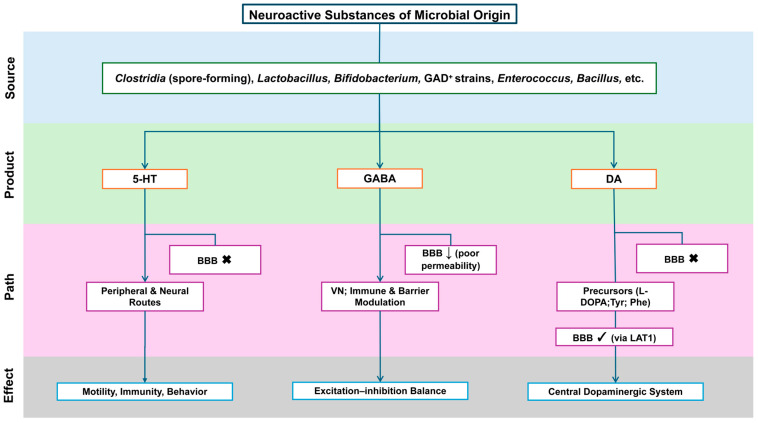
**Neuroactive substances of microbial origin: sources, routes, and CNS reachability. Flow Explanation**: The figure outlines the stepwise flow of microbial–neural interaction from origin to functional outcome. Specific gut bacterial taxa (Clostridia, Lactobacillus, Bifidobacterium, Enterococcus, Bacillus) generate neuroactive products such as 5-HT, GABA, and DA. These compounds differ in their permeability across the BBB and thus utilize distinct communication routes. **5-HT** primarily acts through peripheral and vagal neural pathways to regulate motility and behavior, as it cannot cross the BBB. **GABA** has poor BBB permeability and therefore exerts its effects indirectly—through vagus nerve signaling, immune modulation, and barrier interactions—rather than direct diffusion into the brain. **DA** precursors (L-DOPA, Tyr, Phe) can traverse the BBB through LAT1, integrating into the central dopaminergic system. Collectively, these pathways illustrate how microbial metabolites—through neural, immune, and metabolic conduits—contribute to the MGBA implicated in ASD pathophysiology. **Abbreviations:** 5-HT, 5-hydroxytryptamine (serotonin); GABA, γ-aminobutyric acid; DA, dopamine; BBB, blood–brain barrier; CNS, central nervous system; VN, vagus nerve; L-DOPA, L-3,4-dihydroxyphenylalanine; Tyr, tyrosine; Phe, phenylalanine; LAT1, L-type amino acid transporter 1; GAD^+^, glutamate decarboxylase–positive (GABA-producing) strains. **Symbols**: “×”, cannot cross the BBB; “↓”, low BBB permeability; “√”, BBB-permeant (via LAT1 where indicated).

The gut microbiota both synthesizes neuroactive molecules and modulates their host production through substrate supply and metabolic regulation [39]. Figure 2 outlines key microbial sources, products, BBB permeability, and signaling routes.

**Serotonin (5-HT).** About 90–95% of total 5-HT is synthesized by intestinal enterochromaffin (EC) cells, and peripheral 5-HT does not cross the BBB. Spore-forming microbes promote gut-derived 5-HT synthesis and release by regulating EC cells, influencing motility, immunity, and behavior through peripheral and neural pathways [73,74,75,76,77].

**γ-Aminobutyric acid (GABA).** *Lactobacillus*, *Bifidobacterium*, and some *Bacteroides* produce GABA, whose central effects are likely mediated indirectly—via the vagus nerve, immune signaling, or barrier modulation—rather than by direct BBB passage [78].

**Glutamate and dopamine-related signaling.** Certain strains convert glutamate to GABA, shaping the peripheral substrate balance between excitation and inhibition. Peripheral dopamine poorly crosses the BBB, but its precursors—levodopa (L-DOPA) and large neutral amino acids (tyrosine, phenylalanine)—enter via large neutral amino acid transporter 1 (LAT1), influencing central dopaminergic activity [79,80].

From an interventional standpoint, targeting measurable metabolites or pathways may be more practical than manipulating the microbiota as a whole—for example, enhancing SCFA production through fiber intake or modulating 5-HT precursor availability via strain selection and nutrition. These approaches remain under study, and no definitive human evidence yet supports their efficacy for core ASD symptoms [16].

### 4.3. Tryptophan Metabolism: Imbalance and Neuroinflammation

Tryptophan is metabolized through three main routes: the serotonin, kynurenine (KYN), and indole pathways [47]. Inflammation-induced activation of indoleamine 2,3-dioxygenase (IDO) and tryptophan 2,3-dioxygenase (TDO) shifts metabolism toward the KYN pathway, generating metabolites such as quinolinic acid and KYN, which exert N-methyl-D-aspartate receptor-related neurotoxic and immunomodulatory effects [47,81].

In ASD, peripheral tryptophan (TRP), KYN levels, and their ratios (e.g., KYN/TRP) show inconsistent findings. Although some studies report KYN-pathway activation alongside low-grade inflammation, recent systematic reviews and meta-analyses have not confirmed consistent alterations in peripheral TRP metabolism or tryptophan catabolites. Thus, KYN-pathway imbalance remains a plausible but non-universal mechanism, potentially amplifying gut–immune–brain signaling in subsets of individuals [81,82,83].

## 5. MGBA Dysregulation in ASD: Clinical Relevance and Pathophysiology

### 5.1. Dysbiosis in ASD: Evidence, Confounders, and Variability

Many studies report gut microbiota differences between individuals with ASD and neurotypical controls [2], yet cross-study consistency remains limited. Some cohorts note phylum- or genus-level shifts—such as altered Firmicutes-to-Bacteroidetes ratios [84] and increased *Proteobacteria* or *Sutterella*—while others fail to replicate these patterns [2,85]. No single, universal “ASD microbiome signature” has been identified [86], and results depend heavily on sample source and methodology [87,88,89]. Table 2 summarizes taxa-level findings in ASD, highlighting recurrent trends, inconsistent results, and key confounders across studies.

Drivers of divergence include:

(1) Clinical and behavioral heterogeneity: ASD phenotypes and the spectrum of GI comorbidities vary widely, with pronounced subgroup differences [53].
(2) Lifestyle and confounding factors: notably restrictive diets and food selectivity, which can shape microbial differences and correlate with ASD features [86,89].
(3) Sequencing and analytic variation: 16S rRNA gene amplicon sequencing versus whole-metagenome shotgun sequencing differ in taxonomic resolution and functional inference; batch effects, often addressed with tools like ComBat-Seq or RUV-III-NB, and statistical pipelines, which must account for the compositional nature of microbiome data using methods such as centered/additive log-ratio transformations, ANCOM-BC, or ALDEx2, further influence differential-abundance conclusions [89,106,107,108].

Against this backdrop, research focus is shifting from identifying specific taxa toward mapping functional pathways and metabolic networks. Multi-omics integration suggests that reproducible ASD-associated signals often involve amino acid, carbohydrate, and lipid metabolism, correlating with host transcriptomic and dietary profiles. These findings are largely correlational and remain mechanistic rather than causal [108].

Large-scale, multi-domain metagenomic studies employing machine learning have identified discriminative microbial and functional features—spanning bacteria, archaea, fungi, viruses, and pathways—with within-cohort classification AUCs up to ~0.9. However, external validation, cross-cohort reproducibility, and clinical translation remain major challenges [109].

### 5.2. Barrier Disruption and Neuroimmune Activation

Dysbiosis and impaired epithelial or BBB integrity are candidate mechanisms linking gut and brain in ASD. Loss of mucosal homeostasis allows translocation of bacterial components (e.g., LPS) and metabolites (e.g., *p*-cresol) into the circulation, eliciting systemic inflammation that can influence BBB permeability and central immune activity [110]. Elevated urinary *p*-cresol has been repeatedly reported, though its causality and diagnostic value remain uncertain [111].

A Neuroimaging and biofluid studies further indicate neuroimmune activation: increased 18-kDa translocator protein binding and higher inflammatory mediators in cerebrospinal fluid have been observed, albeit inconsistently and with limited specificity [112,113,114].

Collectively, these findings support a plausible cascade—gut inflammation → barrier dysfunction → systemic cytokines → BBB alteration → glial activation—potentially linking gastrointestinal and behavioral phenotypes in subsets of individuals with ASD. Prospective, controlled studies integrating dietary, pharmacologic, and omic variables are required to determine causality and modifiability [108].

## 6. From Correlation to Causation: Methodological Challenges and Emerging Advances

### 6.1. Core Challenge: Correlation Is Not Causation

Current evidence consistently demonstrates associations among ASD, gut microbiota composition, gastrointestinal symptoms, and behavioral phenotypes. Yet, most studies are cross-sectional or retrospective and therefore cannot infer causality [75]. A central confounder is selective and hypersensitive eating: many children with ASD exhibit sensory aversions to taste, texture, and smell, leading to restricted diets and nutrient imbalances [53]. Such dietary patterns alone can reshape gut microbial and metabolic profiles [86]. Hence, observed microbial alterations may reflect secondary effects rather than etiologic drivers. Robust causal inference will require designs that rigorously control for diet, medication use, and comorbidities [89,115].

### 6.2. Mendelian Randomization (MR) and Multi-Omics Integration

MR uses genetic variants robustly associated with an exposure (e.g., microbial taxa or functions) as instrumental variables to infer causal effects under specific assumptions, thereby mitigating confounding and reverse causation [116,117]. Recent two-sample and bidirectional MR analyses have reported directional links between selected taxa (e.g., *Turicibacter*, *Prevotellaceae*) and ASD, although directions and effect sizes remain inconsistent across studies; some even suggest potentially protective associations [117]. These findings are constrained by weak instruments in microbiome GWAS, horizontal pleiotropy, and sample overlap, necessitating sensitivity analyses (e.g., weighted median, MR-Egger regression, MR-PRESSO, Steiger tests) and colocalization to reduce bias. Overall, MR currently offers preliminary genetic evidence rather than definitive causal proof [117,118].

Multi-omics integration—including metagenomics, transcriptomics, metabolomics, and host phenotypic data—shifts focus from individual taxa to functional pathways [108]. Large-scale integrative studies highlight alterations in amino acid, carbohydrate, and lipid metabolism, correlating with host brain-region transcriptomic profiles and dietary diversity [108]. However, these associations remain primarily correlational and require prospective and interventional validation.

Emerging analytical frameworks such as two-step mediation MR can decompose putative causal chains (e.g., “microbiota → circulating metabolite → ASD”), while reverse MR (“ASD → microbiota”) helps assess bidirectionality and mediation, informing mechanistic targeting of metabolic and dietary pathways [119].

A credible research trajectory would therefore include: (i) prospective cohorts with harmonized data on diet, medication, and behavior analyzed using longitudinal and cross-lagged models; (ii) multivariable MR with colocalization and multi-omics integration to prioritize causal pathways; and (iii) interventional validation through randomized controlled trials (RCTs) enhancing dietary diversity or targeting metabolites and pathways—explicitly accounting for the bidirectional gut–brain–behavior feedback loop and integrating gut-targeted with behavioral-nutritional strategies [89,108]. This comprehensive approach requires a systematic framework that outlines not only the validation of forward causal pathways (microbiome → ASD), but also the investigation of reverse causation (ASD → microbiome), thereby fully addressing the system’s bidirectional dynamics (see Box 1).

## 7. Interventions Targeting MGBA: Therapeutic Prospects and Challenges

We first summarize representative clinical interventions targeting the MGBA—probiotics/prebiotics and fecal microbiota transplantation (FMT)—highlighting study designs, main findings, and limitations (Table 3).

### 7.1. Probiotics and Prebiotics: Promise and Limits

Given recurrent evidence of MGBA imbalance, probiotics and prebiotics have been evaluated as microbiome-targeted interventions in multiple clinical studies [120,121,122]. Systematic reviews and RCTs indicate that these interventions can alleviate GI symptoms in some children and, for certain strains or consortia, improve selected behavioral measures [120,123,124,125]. Other reviews, however, report inconsistent or nonsignificant effects on core ASD symptoms such as social communication and restricted, repetitive behaviors [105]. Overall, the evidence remains heterogeneous, with small and strain-specific effects influenced by dose, duration, baseline GI symptoms, and diet [126,127].

Methodologically, most studies are limited by small samples, heterogeneous interventions, nonstandardized outcome measures, and short follow-up. Open-label designs are prone to placebo and observer bias, and even RCTs show reduced comparability due to differences in strains, consortia, and baseline GI status [105]. A cautious summary is that evidence supporting improvements in GI symptoms is relatively consistent, whereas effects on core behavioral symptoms remain suggestive [126].

Recognizing ASD heterogeneity [86], the concept of personalized or precision synbiotics—tailoring strain–prebiotic combinations to individual microbiome and symptom profiles—is emerging. Pilot and open-label studies show modulation of microbial function and GI outcomes, with limited behavioral improvement, but large-scale, double-blind multicenter trials with standardized endpoints are lacking. Thus, personalized synbiotics currently represent a translational research direction rather than a clinical therapy [128].

**Table 3:** Clinical interventions targeting the gut–brain axis: key trial highlights.

Intervention	Study Design	Sample Type (*n*, Age)	Duration/Follow-Up	Main Findings	Limitations and Challenges	Reference
Probiotics/Prebiotics	Double-blind RCT	80 children (5–14 y)	12 weeks	Microbial α-diversity increases; shifts in several genera; signals linked to GI symptoms/anxiety	Short intervention; 16S limits species/function; activity predicted, not measured	Novau-Ferré et al. 2025 [129]
	Double-blind RCT	46 preschoolers (18–72 mo; 26 vs. 20)	6 months	EEG coherence changes; correlations with behavior + lower inflammatory markers	Small *n*; wide age; no multiple-testing correction; no stratification by GI/sex	Billeci, L. et al. 2023 [130]
	Double-blind RCT	80 children (5–16 y)	12 weeks	No overall core-ASD improvement; age-stratified benefit (reduced hyperactivity/impulsivity in younger children)	Small *n*; mild baseline severity; non-personalized strains; 12 weeks may be short	Rojo-Marticella, M. et al. 2025 [123]
	Post hoc of Double-blind RCT	35 (3–25 y)	16 weeks	Baseline biomarkers ↔ ASD severity; probiotic group showed symptom improvement	Post hoc; wide age; no healthy biomarker controls; no multiple-testing correction	Sherman, H. T. et al. 2022 [131]
	Crossover RCT	15 males (15–27 y)	14-day washout; 28-day treatment	Adaptive behavior improved; trend toward greater social preference (eye-tracking); no GI outcomes captured	Very small, male-only; short; no GI assessment	Schmitt, L. M. et al. 2023 [132]
	Single-blind RCT	180 children (2–9 y)	3 months	Improvements on selected behavioral domains and constipation/diarrhea	Single-blind; short; parent-reported scales, limited validation	Narula Khanna, H. et al. 2025 [120]
	2-stage pilot RCT	35 (3–20 y)	28 weeks (oxytocin added from week 16)	Probiotic + oxytocin > either alone on clinical measures	Small pilot; wide age; two-stage less robust; parental-report bias	Kong, X. J. et al. 2021 [133]
	Nutritional RCT	30 (ASD + neurotypical controls)	12 weeks	Immune reconfiguration (e.g., IFN-γ ↓, IL-8/MIP-1β ↑); behavior not integrated	Small *n*; immunology-only endpoints; limited link to clinical behavior	Naranjo-Galvis, C. A. et al. 2025 [134]
	Parallel-group Double-blind RCT	43 children (2–8 y)	6 months	QoL and some behavioral measures improved; no change in core-ASD severity	Small *n*; COVID-19 recruitment issues; few females; core scale may be insensitive at 6 mo	Mazzone, L. et al. 2024 [135]
FMT	Multi-center Double-blind RCT	29 children with ASD (2–13 y)	4 months	GI outcomes improved; some behavioral measures improved; taxa shifts (e.g., Collinsella) tracked with outcomes; younger children responded better	Open-label components; small *n*; inconsistent endpoints/timepoints; missing final metagenomic/metabolomic timepoint in some.	Chen, Q. et al. 2024 [136]

**Abbreviations**: RCT, randomized controlled trial; GI, gastrointestinal; ASD, autism spectrum disorder; EEG, electroencephalography; IFN-γ, interferon-gamma; IL, interleukin; MIP-1β, macrophage inflammatory protein-1 beta; QoL, quality of life; FMT, fecal microbiota transplantation; *n*, number of participants; y, years; mo, months.

### 7.2. FMT: Breakthroughs and Caution

As a more potent modality for gut ecosystem remodeling, FMT has shown encouraging but preliminary evidence in ASD. Open-label microbiota transfer therapy studies, such as those by Kang et al., reported improvements in pediatric GI symptoms (≈59%) and reductions in Childhood Autism Rating Scale scores (≈47%) sustained over two years [137]. However, these results derive from small, uncontrolled studies and require confirmation in double-blind RCTs [138,139].

Recently, randomized, double-blind, placebo-controlled, and multicenter trials of oral FMT have emerged, elevating the level of evidence. Overall, findings suggest potential benefits for GI outcomes and certain behavioral measures, though effects vary by scale, time point, and cohort. Larger samples and standardized endpoints are needed to clarify reproducibility, effect size, and durability [27,140,141].

Safety and regulatory oversight warrant particular attention. The U.S. Food and Drug Administration (FDA) has issued safety alerts—most notably in 2019 and 2020— regarding the transmission of multidrug-resistant organisms, opportunistic pathogens (e.g., *Escherichia coli*, *Enterococcus faecium*), and rare cases of severe infections and deaths resulting from donor stool–derived FMT materials [142]. These events underscored the need for systematic donor screening, including comprehensive medical history, stool and blood testing for infectious agents, and specific screening for antimicrobial resistance genes or MDRO carriage. Outside of recurrent *Clostridioides difficile* infection (rCDI)—the only indication currently approved for clinical FMT—such interventions are considered investigational and must adhere to regulatory pathways such as FDA Investigational New Drug applications or equivalent local ethical oversight. At present, approved microbiome-based products—Rebyota (enema formulation) and Vowst (oral spore-based therapy)—are indicated solely for rCDI prevention and are not generalizable to ASD [143]. Before clinical adoption, FMT for ASD should be validated through large, multicenter, double-blind RCTs using standardized donor screening, preparation, delivery, and follow-up protocols, to robustly establish both efficacy and safety [139].

## 8. Conclusions and Future Directions

### 8.1. Summary of Current Evidence

The MGBA represents a conceptual framework for exploring part of the clinical heterogeneity observed in ASD. Gastrointestinal symptoms appear more prevalent among individuals with ASD and are frequently accompanied by shifts in microbiome composition and metabolite profiles, which are broadly consistent with mechanistic hypotheses involving neuroimmune and neuroplasticity pathways. Together, these findings form a convergent yet largely associational body of evidence [144]. However, no reproducible or universal “ASD microbiome signature” has been established across cohorts; reported taxa-level alterations often vary in both direction and magnitude among studies, reflecting substantial methodological and population heterogeneity. The most reproducible signals tend to converge on microbial functional pathways rather than on specific taxa, suggesting that pathway-level features may better capture biologically relevant host–microbe interactions. Causal directionality has not yet been established, and major confounders—including selective eating, medication use, and methodological variability—persist [21,75,108]. A recent large-scale, multi-domain metagenomic study identified functional signatures with potential discriminatory capacity, indicating a step toward translational exploration. Nonetheless, these models remain at an early research stage and require rigorous prospective validation, standardized data collection, and cross-population reproducibility assessment before any clinical application [109].

### 8.2. Contribution and Limitations of This Review

The primary contribution of this review is translating abstract concepts of causal inference and precision-oriented study design into actionable frameworks for the ASD-MGBA field. By synthesizing complex methodological challenges and proposing concrete, actionable frameworks (e.g., the bidirectional causality model in Box 1 and the confounder control strategies in Box 2), this work provides a specific roadmap for future research.

However, this review has limitations. As a narrative review, the selection of studies is not exhaustive and is subject to the authors’ selection bias, unlike a systematic review or meta-analysis, which quantifies all available evidence. The forward-looking proposals, particularly for biomarker cutoffs and patient stratification, are intended as conceptual examples to guide future trial design rather than as definitive clinical guidelines.

### 8.3. Priorities for Future Research

Future investigations should aim to move from correlation toward mechanistic understanding and causal inference. In addition to advancing from association to causation, future research must rigorously address key methodological confounders that may obscure microbiome–behavior relationships (Box 2).

Studies need to link specific microbial metabolites and pathways to neural plasticity through controlled animal and organoid models, complemented by longitudinal, standardized multi-omic human cohorts that rigorously account for diet, medication, and geographic variation. Methodologically, these multi-center studies should model as a random effect and employ leave-site-out validation for robustness, while multi-omics integration can be achieved using frameworks such as MOFA+ or DIABLO. MR analyses should be employed cautiously—only when instrumental variable assumptions are satisfied—and their limitations transparently reported. Therapeutic trials should move beyond a one-size-fits-all approach by integrating gut-targeted, behavioral, and nutritional interventions. A critical next step is to stratify participants based on well-defined clinical and biological phenotypes to identify subgroups most likely to benefit from MGBA-targeted therapies. For instance, future RCTs could prioritize recruitment of specific subgroups, such as individuals with (1) a high burden of GI symptoms, (2) pronounced restrictive eating behaviors or sensory hypersensitivities that directly shape the microbiome, or (3) a recent history of significant microbial disruption, such as exposure to antibiotics or PPIs.

To objectify this stratification, a candidate biomarker panel could be employed for patient screening and monitoring. Such a panel might include (1) microbial metabolites like SCFAs and the tryptophan–kynurenine ratio to assess metabolic function; (2) gut-derived xenobiotics like *p*-cresol, which reflect dysbiotic activity; (3) markers of gut inflammation, such as fecal calprotectin; and (4) key systemic inflammatory cytokines. This approach would enable the development of more precise protocols. For example, a trial might set an inclusion criterion of fecal calprotectin > 50 µg/g to target gut inflammation, or it could involve longitudinal monitoring of the tryptophan-kynurenine ratio at 3- or 6-month intervals to gauge therapeutic response to an immunomodulatory probiotic. These trials must be conducted as preregistered, multicenter RCTs with harmonized endpoints to ensure reproducibility and clinical relevance.

Particular caution and regulatory oversight are essential for FMT, which remains experimental outside of *Clostridioides difficile* infection. Ultimately, reproducibility, transparent data sharing, and formal causal inference frameworks will determine whether functional microbiome and metabolite patterns can be validated as reliable biomarkers or as modifiable contributors to ASD-related phenotypes.

## Figures and Tables

**Table 1:** Key microbiota-derived metabolites in the gut–brain axis and their mechanisms.

Metabolites	Primary Sources	Principal Functions and Mechanisms	Potential Effects on Neural Function and ASD
SCFAs	Fermentation of dietary fibre/resistant starch	**Gut barrier**: colonocyte fuel; maintain integrity [24]. **BBB**: support integrity [52]. **Neural**: modulate neurotrophic factors, neurogenesis [52].	Butyrate improves gut/BBB integrity; deficiency may exacerbate neuroinflammation [24].
Neuroactive compounds and precursors	Microbial synthesis; host-microbiota regulation	**5-HT**: microbiota regulate EC-cell synthesis; affect mood/cognition/digestion. **GABA**: produced by select strains; influence brain signaling; promote relaxation/emotional balance. **Dopamine**: tune precursor metabolism; indirect brain effects.	Dysbiosis may reduce 5-HT and GABA synthesis, correlating with ASD behaviors [53].
Kynurenine pathway metabolites	Host TRP metabolism via IDO/TDO	Kynurenic acid (neuroprotective) and quinolinic acid (neurotoxic at high levels).	Dysbiosis/inflammation bias toward neurotoxic quinolinic acid, amplifying neuroinflammation; implicated in ASD pathophysiology [47].
Indole derivatives	Bacterial TRP catabolism	Indole, indole-3-acetic acid; key to gut health.	Urinary/fecal indole derivatives altered in ASD; associated with behavioral symptoms [54].
*p*-Cresol	Microbial metabolism	Neurotransmitter-degradation related; crosses gut barrier and BBB.	Urinary/fecal levels elevated in ASD; linked to increased gut permeability; potential biomarker [55].

Abbreviations: SCFAs, short-chain fatty acids; BBB, blood–brain barrier; 5-HT, 5-hydroxytryptamine (serotonin); GABA, γ-aminobutyric acid; TRP, tryptophan; IDO, indoleamine 2,3-dioxygenase; TDO, tryptophan 2,3-dioxygenase; EC-cell, enterochromaffin cell; ASD, autism spectrum disorder.

**Table 2:** Gut microbiota changes in ASD: summary of consistent and inconsistent findings.

Taxa	Trend in ASD	Evidence Sources	Notes/Confounders
Proteobacteria (phylum)	Commonly increased	[90,91,92,93,94,95,96,97,98,99]	Typically associated with inflammation and disease states.
Actinobacteria (phylum)			
*Sutterella* (genus)			
Firmicutes/Bacteroidetes ratio	Altered, direction inconsistent	[84,93,100]	Typically associated with inflammation and disease states [86].
*Clostridium* (genus)	Mixed findings		
*Prevotella* (genus)			
*Akkermansia* (genus)		[93,101,102,103,104]	
*Bacteroides* (genus)			
*Bifidobacterium* (genus)			May increase after probiotic interventions [105].
*Lactobacillus* (genus)			

**Abbreviations:** ASD, autism spectrum disorder.

## Data Availability

No new data were created or analyzed in this study.

## Abbreviations

5-HT	Serotonin (5-hydroxytryptamine)
ASD	Autism spectrum disorder
BBB	Blood–brain barrier
EC	Enterochromaffin (cells)
ENS	Enteric nervous system
FDA	United States Food and Drug Administration
FMT	Fecal microbiota transplantation
GI	Gastrointestinal
GABA	γ-Aminobutyric acid
HPA	Hypothalamic-pituitary-adrenal (axis)
IDO	Indoleamine 2,3-dioxygenase
IL-1β	Interleukin-1β
IL-18	Interleukin-18
KYN	Kynurenine
LAT1	Large neutral amino acid transporter 1
L-DOPA	L-3,4-dihydroxyphenylalanine
LPS	Lipopolysaccharide
MGBA	Microbiota–gut–brain axis
MR	Mendelian randomization
MR-Egger	Mendelian randomization-Egger (regression)
NMDA	N-methyl-D-aspartate
NLRP3	NLRP3 inflammasome
RCTs	Randomized controlled trials
rCDI	Recurrent Clostridioides difficile infection
SCFAs	Short-chain fatty acids
SNS	sympathetic nervous systems
TRP	Tryptophan
TRYCATs	Tryptophan catabolites
TSPO	18-kDa translocator protein
VN	Vagus nerve
